# Analysis of risk factors to predict communicating hydrocephalus following gamma knife radiosurgery for intracranial schwannoma

**DOI:** 10.1002/cam4.955

**Published:** 2016-11-23

**Authors:** Seunghoon Lee, Seong‐Wook Seo, Juyoung Hwang, Ho Jun Seol, Do‐Hyun Nam, Jung‐Il Lee, Doo‐Sik Kong

**Affiliations:** ^1^ Department of NeurosurgerySamsung Medical CenterSungkyunkwan University School of MedicineSeoulKorea; ^2^ Department of Neurosurgery OrthopedicsSamsung Medical CenterSungkyunkwan University School of MedicineSeoulKorea

**Keywords:** Gamma knife radiosurgery, hydrocephalus, intracranial schwannoma, risk factor

## Abstract

Communicating hydrocephalus (HCP) in vestibular schwannomas (VS) after gamma knife radiosurgery (GKRS) has been reported in the literature. However, little information about its incidence and risk factors after GKRS for intracranial schwannomas is yet available. The objective of this study was to identify the incidence and risk factors for developing communicating HCP after GKRS for intracranial schwannomas. We retrospectively reviewed a total of 702 patients with intracranial schwannomas who were treated with GKRS between January 2002 and December 2015. We investigated patients’ age, gender, tumor origin, previous surgery history, tumor volume, marginal radiation dose, and presence of tumor control to identify associations with communicating HCP following GKRS. To make predictive models of communicating HCP, we performed Cox regression analyses and constructed a decision tree for risk factors. In total, 29 of the 702 patients (4.1%) developed communicating HCP following GKRS, which required ventriculo‐peritoneal (VP) shunt surgery. Multivariate analyses indicated that age (*P* = 0.0011), tumor origin (*P* = 0.0438), and tumor volume (*P* < 0.0001) were significant predictors of communicating HCP in patients with intracranial schwannoma after GKRS. Using machine‐learning methods, we fit an optimal predictive model. We found that developing communicating HCP following GKRS was most likely if the tumor was vestibular origin and had a volume ≥13.65 cm^3^. Communicating HCP is not a rare complication of GKRS for intracranial schwannomas. Under specific conditions, communicating HCP following GKRS is warranted for this patient group, and this patient group should be closely followed up.

## Introduction

Symptomatic hydrocephalus (HCP) resulting from vestibular schwannoma (VS) is frequently observed, especially among patients with an obstruction in their cerebrospinal fluid (CSF) pathway caused by a large tumor 1, 2, requiring ventriculo‐peritoneal (VP) shunt placement 3. However, HCP can sometimes develop without apparent obstruction along the CSF pathways after gamma knife radiosurgery (GKRS) for even small schwannaomas 4, 5. There is little information about the underlying mechanisms that lead to development of communicating HCP following GKRS. One possible contributing factor may be high protein concentration in the CSF 4, 6, 7, 8, 9. In our previous study, we found that large volume tumors and tumors undergoing temporary size changes were correlated with higher incidence of communicating HCP 10.

Unlike plausible complications induced by GKRS, such as damage to the optic apparatus, injury at the pin site, obstructive HCP due to increased tumor size, or various types of radiation injuries, communicating HCP has been easily overlooked as part of the aging process or an inexplicable natural phenomenon, rather than recognizing its role as a possible complication of GKRS. Herein, we attempted to identify the incidence and possible risk factors of communicating HCP after GKRS using our data registry of patients with intracranial schwannomas. Additionally, we applied “machine‐learning” methods to make more accurate predictions of likelihoods for developing communicating HCP following GKRS for intracranial schwannomas. We explored a large panel of machine‐learning approaches for clinicoradiological data from patients who developed communicating HCP. We believe that our investigations reinforce the value of optimal machine‐learning approaches for predictive studies, which helps identify the patients with HCP risk factors before they undergo GKRS.

## Materials and Methods

Since GKRS was introduced into our institution in 2002, we have performed over 8000 GKRSs for a variety of intracranial lesions. A total of 733 patients with intracranial schwannomas were treated with GKRS at our institution between January 2002 and December 2015. We retrospectively reviewed patients’ medical records and magnetic resonance (MR) images with approval from our Institutional Review Board. Among our registry of patients, 31 were excluded because they underwent VP shunt operations before GKRS or they received <4 weeks of follow‐up. We defined HCP as a finding of ventriculomegaly as seen on computed tomography (CT) or MR imaging compatible with clinical manifestations, and requiring a CSF diversion, such as a VP shunt. We evaluated the age, gender, tumor origin, previous surgery history, tumor volume, marginal radiation dose, and presence of tumor control of the 702 eligible patients from our registry. Tumor origins were categorized as vestibular, trigeminal, low cranial nerve (LCN), facial, or cavernous sinus schwannoma. We measured tumor volume with GammaPlan version 9.0 (Elekta, Stockholm, Sweden). We transferred the images to a GKRS planning workstation and three‐dimensional volumetry was conducted using GammaPlan. Tumor control was defined as a reduced or stationary tumor volume after treatment. We regarded any volume change <10% as insignificant change. GKRS was performed by three neurosurgeons (JI Lee, HJ Seol, and DS Kong) at our institution.

### Statistical analyses

Using a Cox proportional hazards model, we performed univariate analyses to identify potential risk factors associated with HCP, and then we conducted multivariate analyses to identify independent risk factors. Results with a *P *< 0.05 were considered statistically significant. All statistical analyses for risk factor verification were performed using SAS version 9.4 (SAS Institute, Cary, NC) and IBM SPSS Statistics 22 for Windows (SPSS Inc., Chicago, IL).

### Machine*‐*learning methods

We investigated a large panel of machine‐learning approaches for clinicoradiological data from patients who developed communicating HCP. We performed our analyses using Python 2.7.3 (Python Software Foundation, Beaverton, OR, USA) with the scikit‐learn, Matplotlib, SciPy, and NumPy packages. We evaluated eight classification methods (nearest neighbors classifier, support vector classifier (SVC), decision tree, Random forest classifier (RF), AdaBoost Classifier, Gaussian naive Bayes (GNB), linear discriminant analysis (LDA), and gradient boosting (GB)) for their predictive performance and stability against data perturbation. Validations were done via three‐fold stratified cross‐validation with a small subset of the dataset. We used the “rpart” package with R 3.1.0 (Vienna, Austria; http://www.R-project.org/) to produce a diagram that illustrates our decision tree. The decision tree, including determining the cutoffs for each variable such as age of a patient, was built with the recursive partitioning analysis. The cutoff splits the data into two disjoint sets as it minimizes the risk prediction (exponential splitting). Also, we estimated the event‐free survival curve for each group using the Kaplan–Meier method.

## Results

Among the 702 enrolled patients, the female‐to‐male ratio was 401:301 and the mean age was 51.5 years (range: 7–84 years). The 568 patients with newly diagnosed schwannomas were treated with GKRS alone and the 134 patients with remnant or regrowing tumors were treated with GKRS as an adjuvant treatment following previous surgical resection. The latter group included cases with regrowing tumors after microsurgery and cases that received GKRS immediately after planned functional preservation surgery 11. Patients were classified according to their tumor origin site: vestibular (571, 81.3%), trigeminal (61, 8.7%), LCN (46, 6.6%), facial nerve (9, 1.3%), or cavernous sinus schwannomas (15, 2.1%). The pre‐GKRS mean tumor volume was 3.6 cm^3^ (range: 0.03–44.1 cm^3^). For 682 patients treated with a single fraction of GKRS, the mean marginal dose was 12.5 Gy (range: 9–20 Gy). The remaining 20 patients, who were treated with fractionated irradiation, had a mean marginal dose of 19.0 Gy (range: 14–22 Gy) administered across 4–5 fractions. Tumor control was achieved in 99.8%, 98.6%, 95.4%, and 76.0% of the patients at 1‐year, 3‐year, 5‐year, and 10‐year follow‐up.

**Figure 1:**
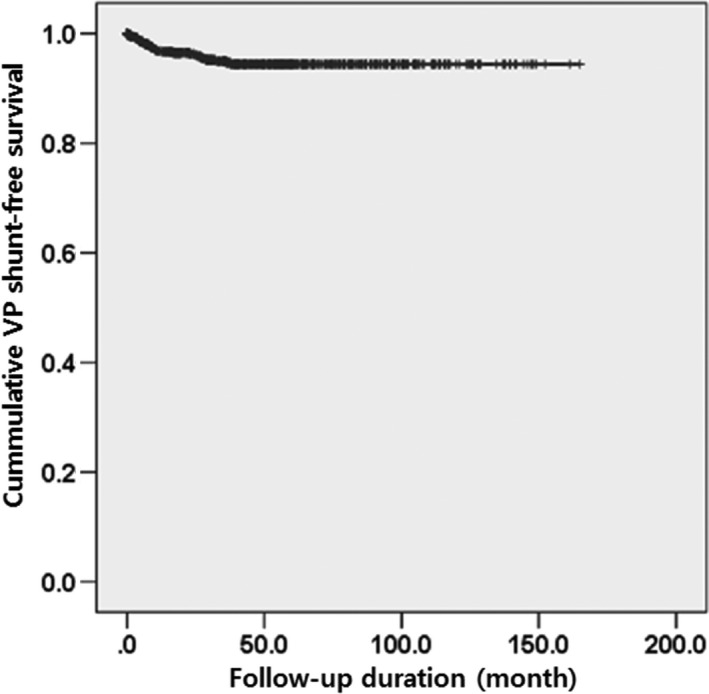
VP shunt‐free survival in patients with intracranial schwannoma after GKRS. A total of 29 of the 702 patients (4.1%) developed symptomatic communicating HCP and required VP shunt placement. The mean duration from GKRS to VP shunt placement was 13.8 months (range: 31 days to 37.8 months). VP, ventriculo‐peritoneal; GKRS, gamma knife radiosurgery; HCP, hydrocephalus.

Twenty‐nine of the 702 patients (4.1%) developed symptomatic communicating HCP and required placement of a VP shunt (Fig. 1). The mean follow‐up duration was 46.5 months, ranging from 29 days to 164.9 months (Table 1). With regard to tumor origin, we divided the patients into VS and non‐VS groups. Univariate analyses revealed that age (*P* = 0.0041) and tumor volume (*P *< 0.0001) were significant HCP risk factors after GKRS on intracranial schwannoma, whereas gender (*P *= 0.2369) and tumor origin (*P *= 0.0641) were not significant prognostic factors. Multivariate analyses showed that all factors except gender were significantly associated with HCP after GKRS: age HR = 1.052, 95% CI: 1.020–1.083, *P* = 0.0011; tumor origin HR = 7.852, 95% CI: 1.059–58.235, *P* = 0.0438; and tumor volume HR = 1.142, 95% CI: 1.096–1.190, *P *< 0.0001 (Table 2).

**Table 1:** Characteristics of patients with intracranial schwannoma after GKRS

Total number of patients	702
F:M	401:301
Mean age (years)	51.5 (7–84)
Origin of schwannoma (%)	
Vestibular	571 (81.3)
Trigeminal	61 (8.7)
Low cranial nerve	46 (6.6)
Facial nerve	9 (1.3)
Cavernous sinus	15 (2.1)
Mean tumor volume (cm^3^)	3.6 (0.03–44.1)
Mean marginal dose (Gy)	
Single GKRS (*n* = 682)	12.5 (9–20)
Fractionated (*n* = 20)	19 (14–22) in 4–5 fractions
Radiological tumor control rate (%)	
at 1‐year f/u	99.8
at 3‐year f/u	98.6
at 5‐year f/u	95.4
at 10‐year f/u	76.0
Mean f/u duration (months)	46.5 (1.0–164.9)
Hydrocephalus (%)	29 (4.1)

GKRS, gamma knife radiosurgery; f/u, follow‐up.

**Table 2:** The analysis of risk factors for hydrocephalus after GKRS on intracranial schwannoma

	Univariate analyses		Multivariate analyses	
	HR (95% CI)	*P*‐value	HR (95% CI)	*P*‐value
Gender (M/F)	0.622 (0.283–1.366)	0.2369	0.460 (0.198 – 1.069)	0.0710
Age (Year)	1.045 (1.014–1.076)	***0.0041***	1.051 (1.020 – 1.083)	***0.0011***
Tumor origin (VS/non‐VS)	6.579 (0.896–48.340)	0.0641	7.852 (1.059–58.235)	***0.0438***
Tumor volume (1/1000 cm^3^)	1.103 (1.065–1.143)	***<0.0001***	1.142 (1.096–1.190)	***<0.0001***

Among the 29 patients who developed HCP after GKRS, 28 patients had VS and 1 patient had a cavernous sinus schwannoma. The mean time between undergoing GKRS and VP shunt placement was 13.8 months (range: 31 days to 37.8 months). The mean age of these patients was 57.9 years (range: 34–81 years). The mean tumor volume was 8.2 cm^3^ (range: 1.3–44.1 cm^3^). Tumor control was achieved in 96.6%, 92.8%, and 81.9% at 1 year, 3‐year, and 5‐year follow‐ups in this patient group.

### Computational classification method

**Figure 2:**
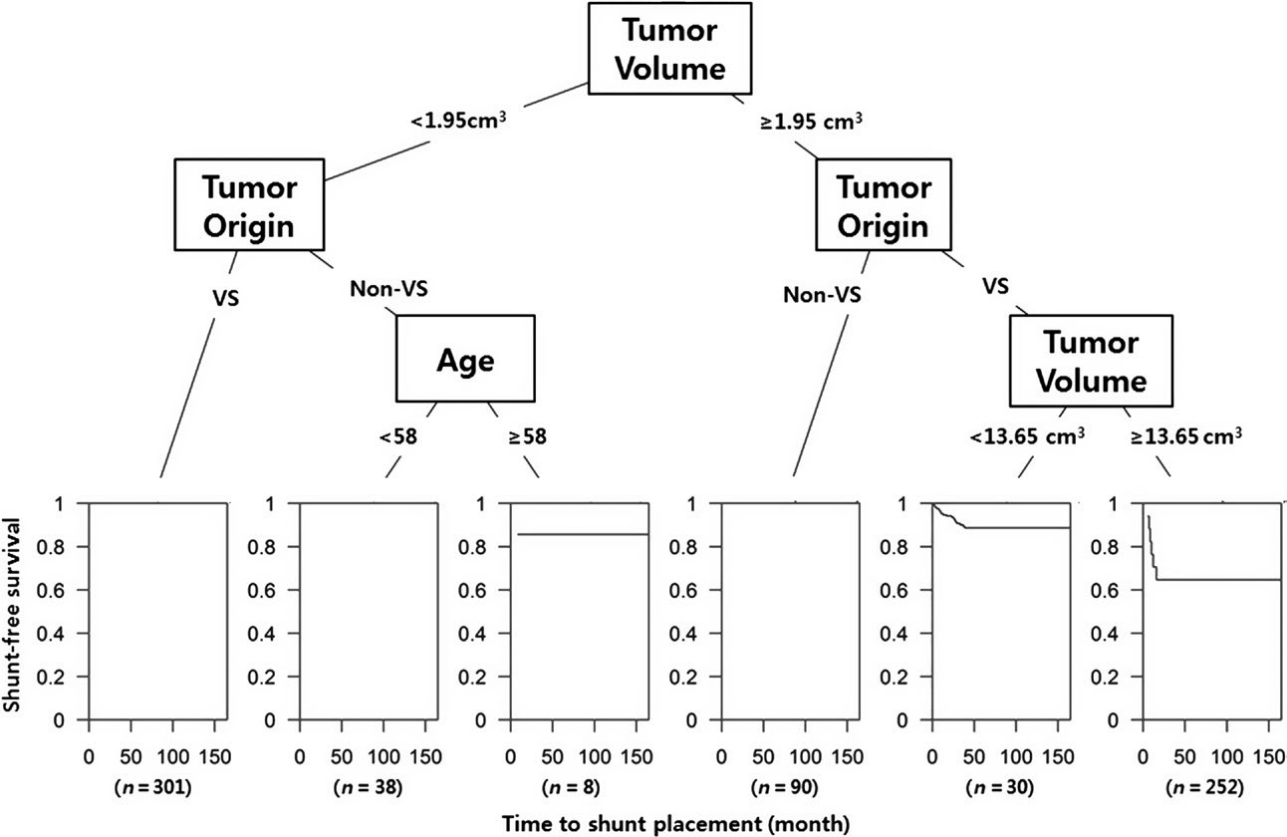
A decision tree for predicting communicating HCP development in patients with intracranial schwannoma after GKRS: Vestibular nerve origin tumor with the volume ≥13.65 cm^3^ was the highest‐risk profile for developing HCP in intracranial schwannoma after GKRS.HCP, hydrocephalus; GKRS, gamma knife radiosurgery.

**Figure 3:**
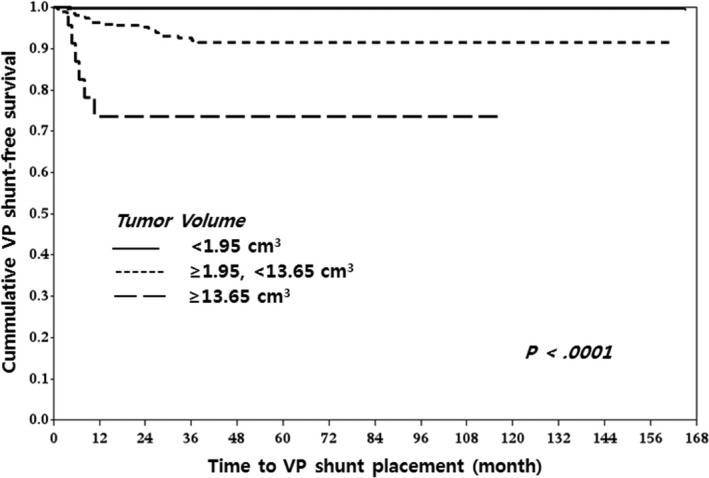
VP shunt‐free survival in each tumor volume group was <1.95 cm^3^, ≥1.95 cm^3^, <13.65 cm^3^, and ≥13.65 cm^3^. There was a significant difference in VP shunt‐free survival curves among three tumor volume groups (*P* < 0.0001); thus, the bigger the intracranial schwannoma, the lower the chance of VP shunt‐free survival after GKRS. VP, ventriculo‐peritoneal; GKRS, gamma knife radiosurgery.

**Figure 4:**
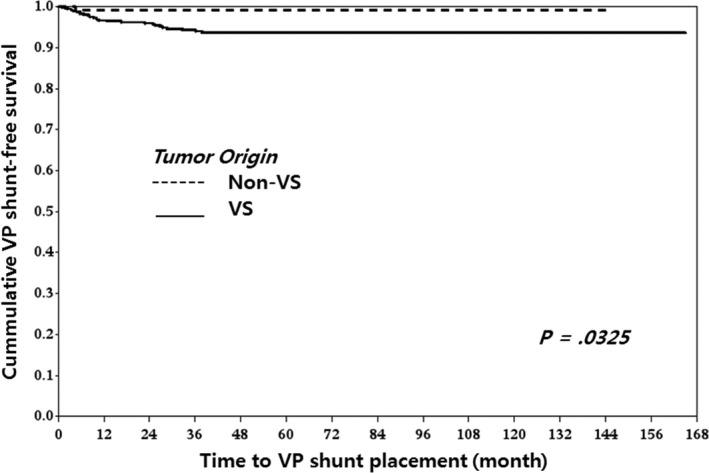
VP shunt‐free survival according to tumor origin: There was a significant difference in VP shunt‐free survival curves between vestibular schwannoma (VS) and non‐VS. (*P* = 0.0325). The patient with VS had a greater risk of developing communicating HCP after GKRS than the patient with non‐VS. VP, ventriculo‐peritoneal; VS, vestibular schwannoma; HCP, hydrocephalus; GKRS, gamma knife radiosurgery.

**Figure 5:**
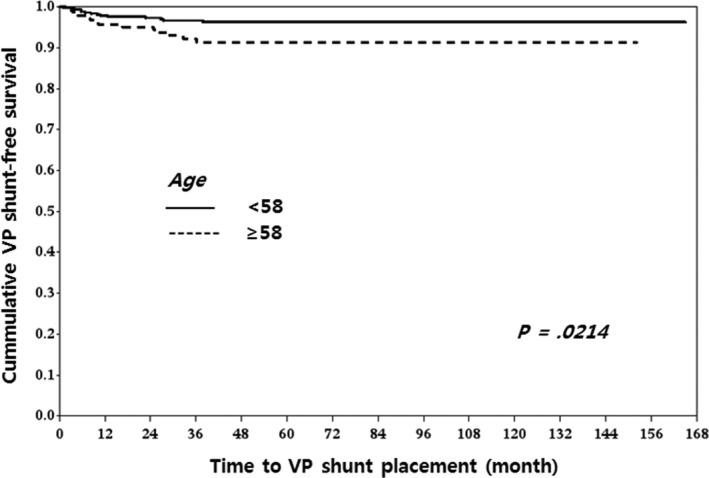
VP shunt‐free survival according to age: There was a significant difference in VP shunt‐free survival curves between patients <58 years old and patients ≥58 years old (*P* = 0.0214). Older patients had a higher risk of developing HCP after GKRS in intracranial schwannoma than younger patients. VP, ventriculo‐peritoneal; HCP, hydrocephalus; GKRS, gamma knife radiosurgery.

Classifications were performed with nearest neighbors classifier, support vector classifier (SVC), decision tree, random forest classifier (RF), AdaBoost classifier, Gaussian naive Bayes (GNB), linear discrimination analysis (LDA), and gradient boosting (GB). There were no significant differences in performance between each classifier as measured by cross‐validation accuracy (Table 3). To identify the decision candidates associated with the highest risks for communicating HCP following GKRS, we drew a diagram based on our decision tree analyses. Decision tree analyses using tumor volume, tumor origin, and patients’ age demonstrated that tumor volume ≥1.95 cm^3^ had the greatest influence on developing communicating HCP. The next most influential factor was tumor origin and, finally, patients with VS and a tumor volume ≥13.65 cm^3^ had the highest risk for developing communicating HCP after GKRS. Patients with VS and a tumor volume between 1.95 and 13.65 cm^3^ also had increased risk. For patients with tumor volumes <1.95 cm^3^ and no VS, being ≥58 years old was an important risk factor for developing HCP (Fig. 2). VP shunt‐free survival curves according to tumor volume (<1.95 vs. ≥1.95 cm^3^ and <13.65 vs. ≥13.65 cm^3^), tumor origin (VS vs. non‐VS), and age (<58 vs. ≥58 years) are depicted in Figures 3, 4, and 5, respectively.

**Table 3:** Results from five‐fold accuracy cross‐validation

Classifier	Accuracy (mean)	SD
Nearest neighbors	0.95	0.02
Super vector classifier	0.96	0.02
Decision tree	0.95	0.03
Random forest classifier	0.95	0.02
AdaBoost classifier	0.95	0.02
Gaussian naive Bayes	0.47	0.06
Linear discrimination analysis	0.96	0.02
Gradient boosting	0.94	0.03

SD, standard deviation.

## Discussion

### Incidence and pathogenesis of HCP in intracranial schwannoma patients

VS‐associated HCP has been reported in many studies, with an incidence ranging from 3.7 to 23.5% 9, 10. Meanwhile, HCP in non‐VS patients is not common and has only been reported in a few studies with very small patient samples 6, 12, 13. We observed a total of 29 (4.1%) communicating HCP patients with intracranial schwannomas after GKRS. Pathogenically, obstructive HCP by a tumor is a mechanical obstruction of the CSF pathway and surgical tumor resection can relieve HCP. However, communicating HCP, which is typically found in cases of intracranial schwannoma after GKRS, is thought to have multifactorial etiologies. Although no consensus explanation has been reached, many hypothetical mechanisms for HCP have been proposed, including plugging by arachnoid granulation due to tumor cells or protein shedding by a tumor resulting in malabsorption, high fibrinogen concentration in the CSF, alterations of CSF flow dynamics in the basilar cisterns, decreased intracranial compliance due to adhesion of the subarachnoid space, tumor‐induced arachnoiditis, or meningeal adhesions due to recurrent tumor bleeding 4, 5, 10, 14, 15, 16, 17.

### Protein concentration in CSF and tumor size

High protein concentration in the CSF has been widely considered to be the main contributing factor for HCP and many studies have shown elevated intraventricular CSF protein concentrations 1.6–15 times above normal levels in HCP cases 6, 14, 16, 18. Tumor size is also considered to be an important factor for developing HCP in VS patients after GKRS in the same context. The positive correlation between CSF protein and tumor size indicates that tumor enlargement is a contributor to increased CSF proteins 6, 14, 19, 20, which is supported by our previous study 10. We observed that all events requiring shunt placements for HCP occurred within 3–4 years after GKRS, during which time tumor volume expansions occurred. However, the role of CSF protein levels and tumor size was discordant in other studies; the highest CSF protein concentrations were found in patients with the smallest tumors and middle‐range concentrations were found in patients with the largest tumors 6. In this study, we also observed that smaller‐tumor cases were not totally free from HCP development. Thus, CSF protein concentration and tumor size do not seem to play an independent role in communicating HCP development after GKRS in isolation.

### Age

Aging has also been investigated as a contributing factor for HCP in cases of intracranial schwannoma after GKRS. Several studies have demonstrated that VS patients with HCP are older than VS patients without HCP. And the possibility remains that normal pressure HCP was not totally excluded and overlapped somewhat in these studies. In our study, we found a significant statistical relationship with age in multivariate analyses, although tumor volume was a more potent influencing factor on communicating HCP development after GKRS, as illustrated in our decision tree (Fig. 2). We explain this mechanism as an aging‐related decrement in the reserve capacity of CSF absorption 12, 21, 22. However, other published studies have failed to find a similar positive correlation with aging 7, 16, 17.

### Tumor origin

Among the 29 HCP patients with intracranial schwannomas, there was only one patient who had HCP and no VS after GKRS. In another study, the authors analyzed 14 patients (5.7%) with HCP after GKRS, and three of them were non‐VS patients 12. This paucity of communicating HCP after GKRS in non‐VS patients draws our attention to tumor origin, which corresponds to the location of the VS in the internal auditory canal in the cerebellopontine (CP) cistern. The surgical anatomy of the CP angle is complex and the CP cistern, where the cranial nerve VIII is located, is crowded by several vulnerable cranial nerves and small vessels directed toward the brainstem and the cerebellum. The CSF in the CP cistern is directly drained from the 4th ventricle through the foramen of Luschka. Stagnation of CSF flow in the CP cistern caused by a tumor and its products may have a substantial effect on CSF dynamics and may theoretically result in HCP due to there being very small spaces to redistribute the CSF from the fourth ventricle. Advances in MR imaging techniques have enabled us to understand more about CSF dynamics, and we plan to accumulate more evidence regarding the contribution of tumor location to developing HCP among VS patients after GKRS in future studies.

### Risk stratification of HCP development in patients with intracranial schwannoma

To the best of our knowledge, we could not find any significant risk stratification of developing communicating HCP after GKRS in patients with intracranial schwannoma. Given the most probable and statistically significant risk factors we identified, we propose the evidence should be used to plan treatment strategies for intracranial schwannoma patients who may undergo GKRS. As shown in our decision tree analysis (Fig. 2), tumor volume and age at the time of GKRS further divides patients into three risk subgroups: high‐risk group with VS and tumor volume ≥13.65 cm^3^; intermediate‐risk group without VS, tumor volume <1.95 cm^3^, and patient age ≥58 years; and low‐risk group without VS and tumor volume ≥1.95 or <13.65 cm^3^. For patients with high risk for developing HCP, short‐term follow‐up with MR imaging and close monitoring of communicating HCP are strongly advised. However, there was only one non‐VS patient who developed hydrocephalus after GKRS, especially among the total of eight patients with <1.95 cm^3^ and age ≥ 58, which could probably exaggerate the risk of developing hydrocephalus in this group. In our study, without this exceptional patient, we might have concluded that non‐VS tumor had less possibility of developing hydrocephalus after GKRS.

We made a practical predictive model for risk of developing communicating HCP among a large cohort of patients with intracranial schwannoma after GKRS. Communicating HCP in VS patients following GKRS was not a rare event in a selected group of patients. VS patients with tumor volumes ≥13.65 cm^3^ had the highest‐risk profile for developing HCP from intracranial schwannoma after GKRS. Therefore, communicating HCP following GKRS is warranted for this patient group, and this patient group should be closely followed up.

## Conflict of Interest

The authors have no disclosures to make.
